# Cerebral salt wasting syndrome in an elderly patient with cerebral small vessel disease

**DOI:** 10.1002/ccr3.9404

**Published:** 2024-09-30

**Authors:** Leslie Bahn Kawa, Kulsoom Fatima Bhatti

**Affiliations:** ^1^ Geriatric Medicine Eastbourne District General Hospital, East Sussex NHS Trust Eastbourne East Sussex UK

**Keywords:** cerebral salt wasting syndrome, cerebral small vessel disease, syndrome of inappropriate antidiuretic hormone

## Abstract

Hyponatremia is a common electrolyte disturbance seen among the acute geriatric admissions with two common diagnostic entities; the syndrome of inappropriate antidiuretic hormone (SIADH) and cerebral salt wasting syndrome (CSWS) that have different clinical and biochemical presentations, different pathogenesis and therapeutic approaches. Hyponatremia caused by CSWS in patients with cerebral small vessel disease (cSVD) a prevalent condition among the elderly, can be masked in geriatric patients with concomitant fluctuating neurological deficits. Correct diagnosis is crucial to appropriate management. In this case report we describe an association between hyponatremia caused by CSWS in a patient with cSVD.

## 1 INTRODUCTION

Hyponatremia is one of the commonest electrolyte abnormalities seen among the acute admissions in clinical practices. It is very common among the elderly patients in acute geriatric medical wards^1^, ^2^ and is associated with poor clinical outcomes.^3^

The two commonest causes of hyponatremia are the syndrome of inappropriate antidiuretic syndrome (SIADH) and the cerebral salt wasting syndrome (CSWS). It is difficult to differentiate these two conditions clinically and biochemically because of their similarities. The CSWS is defined as hypovolemic hypo‐osmolar hyponatremia associated with high renal sodium excretion and urine osmolality.^4^, ^5^ It is very common among those patients who sustain cerebral insults and is well documented among those with aneurysmal cerebral subarachnoid hemorrhages.^6^, ^7^ Although, its pathophysiology is not delineated, it is postulated to be due to excess natriuretic peptides secretions from the brain insults^8^ and the reduced sympathetic effect on the juxtaglomerular leading to diuresis and natriuresis.^3^ The SIADH on the other hand is caused by excessive antidiuretic hormone and presents as euvolemic hypo‐osmolar hyponatremia with hypo‐osmolar urine and sodium at least between 20 and 40 mOsmol/L. Nevertheless, early differentiation and diagnosis are very important as their treatments differ.^9^, ^10^

Although, these conditions are common, no case of CSWS is reported in patients with cerebral small vessel disease (cSVD). We, therefore, describe a case of incidental CSWS in an elderly patient with cSVD presenting with fluctuating neurological deficits.

## 2 CASE DESCRIPTION

### 2.1 History

A female in her late 70s who had confusion and declining memory was brought into the emergency department for acute medical admission by her daughter who was concern about her deteriorating conditions. She has had chronic but fluctuating confusion for over a year leading to several prior admissions. She has had no history of head injury, seizure, or syncope and her drug history was unremarkable.

### 2.2 Examination

On this admission, she was noted to be more confused than usual by her daughter and registered an abbreviated mental test score (AMTS) score of 6, although, a formal diagnosis of dementia was not established. She has no other focal neurological deficits but appeared clinically dehydrated with a dry tongue, reduced skin turgor, and a blood pressure of 98/55 mmHg with no evidence of sepsis.

## 3 METHODS (DIFFERENTIAL DIAGNOSIS, INVESTIGATIONS, AND TREATMENT)

Her blood sugar and all others were normal except for an isolated moderate hyponatremia of 123 mmol/L with a calculated serum osmolality of 261 mOsmol/L suggesting a hypovolemic hypo‐osmolar hyponatremia. Provisional diagnosis of delirium secondary to dehydration, hyponatremia secondary to SIADH, and vascular dementia were made.

A casual fluid restriction of ≤1 L daily was imposed in view of SIADH whilst waiting for her urine osmolality and sodium levels from the laboratory.

**FIGURE 1:**
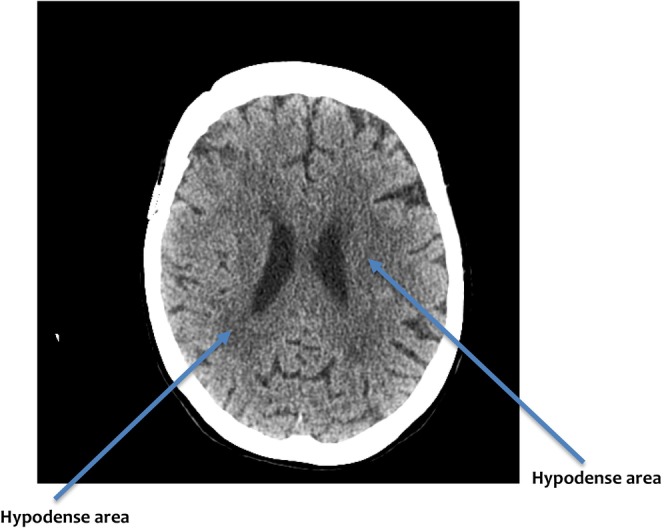
Bilateral periventricular and subcortical low attenuation of white matter changes consistent with moderate cerebral small vessel disease.

A CT head on her second day of admission showed moderate cSVD (Figure 1). She complained of inability to walk and felt glued to her chair despite having normal motor functions. Request to get her to stand and walk was not possible, though, she wanted to follow the command. This lasted for several hours and improved in the evening and fluctuated over the next 2 days of her admission.

Meanwhile, her sodium (122 mmol/L) dropped despite fluid restriction. On the third day of admission, her sodium dropped further to 120 mmol/L and her serum osmolality was 256 mOsmol/L. Her urine osmolality and sodium were 345 mOsmol/L and 54 mmol/L, respectively, suggesting renal sodium loss. Her confusion was objectively better. A revised diagnosis of cSVD complicated by CSWS, apraxia and vascular dementia was made, and she was prescribed 2 L of 0.9% normal saline over 24 h.

## 4 CONCLUSION AND RESULTS

CSWS is a rare cause of hyponatremia associated with cSVD. A cluster of clinical signs and symptoms, biochemical parameters and fluctuating neurological deficits associated with evidence of cSVD on brain imaging can clinch the diagnosis. Treatment is with intravenous saline infusion.

After 24 h of saline infusion, the patient's serum, urinary electrolytes, and osmolality (Table 1) continued to improve, and she was discharged on the fifth day after admission.

**TABLE 1:** Cumulative biochemistry and other investigational results.

Investigations	Daily results						Reference range (mmol)
	Day 1	Day 2	Day 3	Day 4	Day 5	Day 14	
Serum sodium	123	122	120	123	130	128	133–146
Serum potassium	5.3	5.2	5.1	5.0	4.9	5	3.5–5.3
Creatinine	110	100	92	85	80	85	45–84
eGFR (mL/min/1.73 m^2^)	44	50	55	56	60	56	>90
Serum osmolality (mOsmol/Kg)	261	260	256	270	276	279	275–295
Urine osmolality (mOsmol/Kg)			345			320	275–295
Urine sodium			54			50	
Serum uric acid μmol/L	110				115		155–357
Thyroid function tests	Normal						
Chest x‐ray	Normal						
Computer tomography of head (CT)	Moderate cerebral small vessel disease						

She was seen at the ambulatory clinic a week later with her daughter and has been coping well with usual fluctuations of her confusion without apraxia. She was independent of all activities of daily living but needed support from career twice weekly and her daughter live nearby and provides extra support. Her serum sodium was 128 mmol/L, serum osmolality 279 mOsmol/L, and stable urinary sodium and osmolality. She was discharged from the ambulatory clinic for her GP to follow her up in primary practice. Her progressive sodium over 3 months remained stable between 26 and 31 mmol/L.

## 5 DISCUSSION

CSWS is a common cause of hyponatremia among the neurosurgical patients and is well documented among those with aneurysmal cerebral subarachnoid hemorrhages.^6^, ^7^ Although, its pathophysiology is not delineated, it is postulated to be due to excess natriuretic peptides secretions from the brain insults^8^ and the reduced sympathetic effect on the juxtaglomerular leading to diuresis and natriuresis.^3^

CSWS can occur among the geriatric patients who have high prevalences of both hyponatremia^1^ and cSVD^8^ after excluding other common causes of hyponatremia. Hyponatremia caused by the CSWS is predominantly hypovolemic hypoosmolar compared to hyponatremia caused by SIADH where it is euvolemic hypoosmolar hyponatremia. There is debate as to whether these two conditions are two different entities or the variants of the same condition.^12^, ^13^ The former is driven by the natriuretic peptides and the latter by the antidiuretic hormone.^10^ They, however, have relatively similar clinical and biochemical parameters and are often difficult to diagnosed early. Nevertheless, it is imperative to make an early differentiation because the treatments vary with fluid administration in CSWS and restriction in SIADH.

Patients with CSWS usually have a history of brain insult, and this could be a pointer towards the diagnosis. However, many geriatric patients, do not have a history of brain injuries but do have undiagnosed underlying cSVD that can present with hyponatremia and other neurological deficits.^1^, ^11^ The underlying mechanism causing CSWS in these patients is unknown. However, we postulate that persistent microinfarcts from the cSVD in areas controlling the natriuretic peptide homeostasis leads to excessive secretion as it is seen in those with traumatic brain injuries.

The diagnosis of CSWS can be considered in elderly patients who present with typical clinical and biochemical evidences in the background of lack of potential causes of hyponatremia. Additional evidence can be sought from the presence of the cSVD on brain imaging which often associates with the fluctuation of neurological deficits.^14^ This is further supported by the patient's response to intravenous saline infusion rather than restriction.

Several studies^15^, ^16^, ^17^, ^18^ have recommended certain clinical, biochemical criteria for the diagnosis of CSWS. Some of these tests^16^, ^17^ are not readily available in most practices to make a quick diagnosis. We, therefore, propose that an history of fluctuating neurological deficits in the presence of cSVD on brain imaging as supportive parameters for the diagnosis of CSWS in the presence of relevant clinical and biochemical profiles.

## AUTHOR CONTRIBUTIONS

**Leslie Bahn Kawa:** Conceptualization; data curation; formal analysis; methodology; validation; writing – original draft. **Kulsoom Fatima Bhatti:** Data curation; investigation; validation.

## FUNDING INFORMATION

None.

## CONFLICT OF INTEREST STATEMENT

None.

## CONSENT

Written informed consent was obtained from the patient to publish this report in accordance with the journal's patient consent policy.

## Data Availability

The data are not publicly available due to privacy or ethical restrictions.
